# Plant-based index linked to fall risk in older Chinese adults: cross-sectional evidence from a national cohort

**DOI:** 10.1007/s40520-024-02838-z

**Published:** 2024-09-05

**Authors:** Fuli Yang, Junguo Jin, Jieliang Liu, Xiaoqi Lu, Huyi Jiang, Huixin Tan, Fenghua Zhou, Ping Zeng

**Affiliations:** 1 Guangdong Cardiovascular Institute, Guangdong Provincial People’s Hospital, Guangdong Academy of Medical Sciences, Guangzhou, 510080 Guangdong China; 2 Department of Cardiology, Peking University First Hospital, No. 8 Xishiku St, Xicheng District, Beijing, 100034 China; 3 Department of Cardiology, Guangdong Cardiovascular Institute, Guangdong Provincial People’s Hospital (Guangdong Academy of Medical Sciences), Southern Medical University, Guangzhou, 510080 Guangdong China; 4 School of Traditional Chinese Medicine, Southern Medical University, Guangzhou, 510515 China

**Keywords:** Falls, Older population, *Plant-based diet*

## Abstract

**Objectives:**

Epidemiology showed that the falling incidences increased with advanced age, and recent findings found link between nutritional intake and risk of falls. Nevertheless, the relationship between different plant-based diets and the risk of falls in older adults remains unclear. Our investigation aimed to evaluate the correlation between various plant-based diet indices and the occurrence of falls.

**Design:**

This study is a cross-sectional and post-hoc analysis from a national cohort study.

**Setting and participants:**

We included individuals over 65 years from the Chinese Longitudinal Healthy Longevity Survey (CLHLS) recruited in 2018 with information on falls and dietary assessments, finally 11,044 participants were eligible.

**Measurements:**

Using food frequency questionnaire (FFQ), we calculated plant-based index scores categorized as unhealthy plant-based index (uPDI) and healthy plant-based index (hPDI). The primary outcome was falls obtained from questionnaire. Statistical analysis was performed utilizing logistic regression model to investigate the relationship between the plant-based diet indices and falls. We also used the subgroup analysis to investigate the interaction of falls and plant-based diet index (PDI) among different status and used the restricted cubic spline (RCS) curves to investigate the connection between the PDI scores and falls risk.

**Results:**

Among 11,044 participants included in our study, a total of 2493 fall cases were observed. The logistic regression analysis revealed that the plant-based index related to falls. In the adjusted model, per 10-unit increment of hPDI has a significant decreased risk of falls (odd ratio [OR]: 0.85, 95% confidence interval [CI]: 0.79–0.91, P for trend < 0.001) and per 10-unit increment in uPDI increased the risk of falls (OR: 1.21, 95% CI: 1.13–1.30, P for trend < 0.001). We also revealed an interaction between smoking status and falls among the uPDI group (*P*_interaction_ = 0.012). Finally, we found that with plant-based index scores increased, the odds of falls among hPDI decreased (P for overall < 0.001, P nonlinear = 0.0239), and the odds of falls among uPDI increased (P for overall < 0.001, P nonlinear = 0.0332).

**Conclusion and implications:**

We found significant association between the Plant-based diet index and the risk of falls, highlighting the key role of the consumption of nutritious *plant-based* foods on the risk of falls, which needed take into account in developing intervention and prevention strategies to decrease falls among older Chinese adults.

**Supplementary Information:**

The online version contains supplementary material available at 10.1007/s40520-024-02838-z.

## Introduction

From the data of the World Health Organization [1], falls were the second dominant cause of unintentional injury deaths worldwide. Falling was defined as event which a person inadvertently came to rest on lower level, like the ground or floor. Numerous factors were linked to falling incidences, including advanced age, Parkinson’s disease (PD), muscle weakness among others [2]. Epidemic evidence showed that the older individual experienced at least one fall per years, and 10% of those individuals suffering fall-related injuries [3]. Identifying the risk of falls among older adults was crucial due to the significant economic burdens they incurred. In America, the estimated cost reached up to 50 billion dollars annually, while the cost per person per fall ranged from16 to 3812 dollars in China [4, 5]. Therefore, identifying the risk factors of falls were of the utmost importance in preventing falling among older adults.

Diet played vital role in various aspects of health, including frailty, PD, and bone mineral density [6–8]. According to recent research, diet could be divided into distinct components, including dietary nutrition and dietary patterns like Mediterranean diet, which focused on specific nutrients and the overall combination of foods intake. In the past, plentiful studies primarily investigated the relationship between individual nutrients and health outcomes, for instance, deficiencies in calcium and vitamin D contributed to the development of osteoporosis [9]. Recent studies had shifted towards examining comprehensive diet patterns, encompassing the quantity, types and combinations of foods intake, connecting to the risk of many diseases and deaths. Unhealthy diet and malnutrition are among the most critical risk factors for chronic diseases. Thus, an increasing number of dietary patterns are urgently being proposed to prevent the occurrence of chronic diseases [10–12]. Certain dietary patterns were linked to PD, cardiovascular diseases and cognitive decline, which were also factors for falls. In addition, the diet pattern of Mediterranean diet [13] confirmed to be correlated with lower risk of falls. Therefore, the influence of diet on the prevalence of falls should not be overlooked.

Among various diet patterns, the plant-based diet primarily focused on eating foods derived from plants, such as fruit, vegetables, whole grains, gained considerable attention due to its potential benefit to diabetes mellitus (DM), cardiovascular diseases, cognitive impairment [14–18]. The plant-based dietary indices, namely the healthy plant-based diet index(hPDI) and the unhealthy plant-based diet index(uPDI), were form of plant-based diet. Previous review had found that hPDI was associated with reduced risk of cancer, cardiovascular diseases [19, 20]. On the other hand, the uPDI had found not only to be connected with symptoms of depression and anxiety but also linked to decrease risk of all-caused mortality in older individuals [21, 22]. However, there was no study investigating the relationship between the two plant-based diet index and the incidence of falls. Our study was to explored the potential connection between the PDI and falls among the population aged > 65 years. *We hypothesized that adherence to a healthy plant-based diet (hPDI) is associated with a decreased risk of falls*, while an unhealthy plant-based diet (uPDI) is associated with an increased risk of falls in older Chinese adults.

## Methods

### Participants and study design

**Fig. 1:**
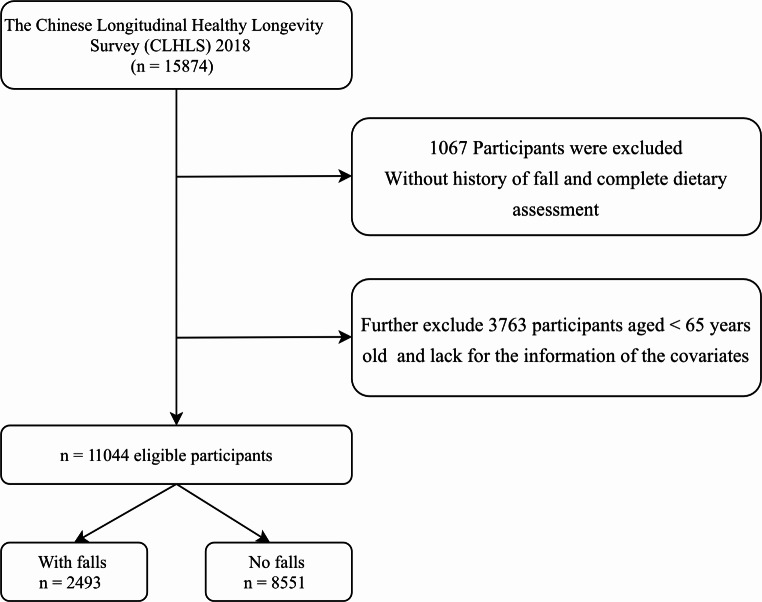
Study flowchart

The data in our study were derived from Chinese Longitudinal Healthy Longevity Survey (CLHLS) which was a longitudinal study. The CLHLS was conducted from 1998 to 2018, focusing on people aged 60 years and above, and follow-up interview were conducted every 3 or 4 years. The sample size was calculated based on the prevalence of falls among older adults in the Chinese population [23], ensuring sufficient power to detect significant associations between the PDI and fall risk. The calculations accounted for potential confounders and aimed to achieve a confidence level of 95% with a power of 80%. Our study recruited 15,874 participants in 2018. We excluded the participants without history of fall and complete dietary assessment (*n* = 1067) and we further excluded the participants aged < 65 years old and lack of the information of the covariates (*n* = 3763). Finally, we conducted a total of 11,044 participants eligible for our post-hoc analysis. The missing values of covariant were removed. The flow chart was provided in Fig. 1.

### Definitions and measurements

Falling was an event which caused a person inadvertently rest on the ground or floor or other lower level. The history of falls was captured through the questionnaire based on self-reporting or kin-reporting. The plant-based diet index developed by Satija et al. has been applied in a variety of studies, and the validity and reliability of the PDI have been confirmed by previous research [24]. Thus, healthy and unhealthy plant-based indices (hPDI and uPDI) are promising methods to quantify plant-based diet adherence and valuable tools [25]. The plant-based diet was defined as intaking more fruit, vegetables, nuts, olive oil, whole grains, beans and limited consumption of dairy, eggs, meat and fish [18]. Our study scored PDI based on frequency of ingestion. In some previous studies, the use of non-quantitative food frequency questionnaires to assess eating patterns has been proven to be reliable and effective. In addition, previous studies have shown that frequency of intake is more important than portion size [26]. We used simplified FFQ to assess each participant’s dietary information. We categorized 16 foods into three groups: healthy plant foods group (whole grain, fresh fruit, vegetable oil, fresh vegetable, legume, garlic, nut, tea), less healthy plant foods group (refined grain, sugar, salt-preserved vegetable), and animal-based foods group (animal fat, meat, fish, egg, dairy products) [16]. The questionnaire had binary options for whole grain, vegetables oil, refined grain, animal fat, including “yes” or “no”. It offered four responses choice to the frequency of consumption for those products (fresh fruit, fresh vegetable, and egg), including “almost every day”, “quite often”, “occasionally”, or “rarely or never”. The questionnaire had five options for legume, garlic, nut, tea, sugar, salt-preserved vegetable, meat, fish, dairy products, including “almost every day”, “≥1 time per week”, “≥1 time per month”, “occasionally”, or “rarely or never”. Using the dietary data, we calculated the scores of hPDI and uPDI. Scores ranging from 1 to 5 were allocated to the intake frequencies of 16 foods groups. For the hPDI, positive scores were assigned to healthy plant-based foods (5 for the most frequent consumption and 1 for the least frequent consumption), while scores for less healthy plant-based foods and animal-based foods were reversed (1 for the most frequent consumption and 5 for the least frequent consumption). For the uPDI, healthy plant-based foods were reverse-scored, while unhealthy plant-based foods and animal-based foods were given positive scores, with the same scoring system as mentioned earlier. We summarized the scores for the 16 foods group for everyone to obtain uPDI and hPDI. The specific information was provided in Table S1.

### Covariates

Covariates included sex (male or female), age (years), ethnic group, place of residence, marital status, alcohol consumption, smoking status, year of schooling, body mass index (BMI), hypertension, DM, heart disease, respiratory disease, stroke/cerebrovascular disease and cancer, which based on participants’ responses to questionnaire. Ethnic group was classified as Han and other. The residence variable was classified as either rural or urban. Marital status was divided into married, co-partnered or single. Participants were classified as smokers or drinkers based on their current or past smoking or drinking habits. The questionnaire asked for the participants’ educational level, expressed as year of schooling. Participants were also asked whether they had history of hypertension, DM, heart disease, respiratory disease (bronchitis, emphysema, asthma or pneumonia), stroke/cerebrovascular diseases and cancer, and those who answered “yes” were considered to have history of the specified illness. During the course of the interview, participants’ weight (in kilograms, kg) and height (in meters, m) were measured by interviewers. BMI was measured as the quotient of body weight divided by the square of height (kg/m^2^).

### Statistical analysis

Quantitative variables were described as means ± standard deviations, and qualitative variables were presented as absolute proportions (%). We applied the logistic regression models to estimate the OR of falls associated with PDI and we calculated *P*-trend to assess the linear trend of OR in logistic regression models, using the median value of each diet score quartile. OR and 95% CI were calculated by quartiles.

In addition, we conducted subgroup analyses to determine whether the association differed by age (< 80 or ≥ 80), sex (Male or Female), ethnicity (Han or Others), smoking status (Current or former/ Never), residence (Urban or Rural), marital status (Married or Single) and health status (With chronic diseases or Without chronic diseases). To evaluate the presence or absence among these factors, we used the *P*-value for interaction as indicators.

We deleted the missing data in the main analysis, and we implemented in the way of random forest to process the missing data in the sensitivity analysis. Twenty imputed datasets were created using multiple imputation with chained equations (MICE) [27] to handle missing values, and the results were combined following Rubin’s rule [28]. The detailed percentages of missing data are shown in Supplementary Table S5 in Supplementary Material.

The relationship between PDIs and the risk of falls was analyzed using multivariable-adjusted restricted cubic spline (RCS) curves to determine the dose-response relationship. To account for the large sample size and balance curve smoothing while avoiding overfitting, we plotted four-knot cubic splines to explore potential non-linearities between PDIs and the risk of falls.

We employed sensitivity analyses to enhance the robustness of results. Initially, we further adjusted the chronic disease (hypertension, heart disease, DM, respiratory disease, stroke/cerebrovascular disease and cancer). In addition, we implemented in the way of random forest to process the missing data.

We conducted all analyses using R (version 4.2).

The manuscript was written in rigorously to compliance with the STROBE statement [29] listed in Table S2.

## Results

### Characteristics of participants

This study included a total of 11,044 participants, among whom 6123 were female, and 4921 were male. A total of 2493 (22.60%) cases of falls were documented. Divided into two groups according to the incident of falls, Table 1 showed the characteristics of the study. Fall group demonstrated higher proportion of female, current or former smokers, lower BMI, lower hPDI scores, fewer years of education, and higher uPDI scores. Additionally, the prevalence of heart disease and stroke or cerebrovascular disease was found to be higher among individuals in the falls group (*P* < 0.05).

**Table 1:** Baseline characteristic

	Overall	Without fall	Fall	*P* value
No. of participants	11,044	8551	2493	
hPDI score	49.95(6.48)	50.18 (6.46)	49.18 (6.46)	< 0.001
uPDI score	49.61 (7.04)	49.36 (7.02)	50.45 (7.05)	< 0.001
Age, years	84.98 (11.75)	84.22 (11.68)	87.57 (11.62)	< 0.001
Gender (%)				< 0.001
Female	6123 (55.40)	4586 (53.60)	1537 (61.70)	
Male	4921 (44.60)	3965 (46.40)	956 (38.30)	
Ethnic group (%)				0.005
Han	10,405 (94.20)	8027 (93.90)	2378 (95.40)	
other	639 (5.80)	524 (6.10)	115 (4.60)	
Residence (%)				0.258
Rural	7815 (70.80)	6074 (71.00)	1741 (69.80)	
Urban	3229 (29.20)	2477 (29.00)	752 (30.20)	
Marital status (%)				< 0.001
Married an co-partnered	6436 (58.30)	4809 (56.20)	1627 (65.30)	
Single	4608 (41.70)	3742 (43.80)	866 (34.70)	
Alcohol consumption (%)				0.170
Never	9437 (85.40)	7285 (85.20)	2152 (86.30)	
Current or former	1607 (14.60)	1266 (14.80)	341 (13.70)	
Smoking status (%)				< 0.001
Never	7695 (69.70)	2667 (31.20)	682 (27.40)	
Current or former	3349 (30.30)	5884 (68.80)	1811 (72.60)	
BMI (kg/m^2^)	22.40 (4.44)	22.50 (4.45)	22.06 (4.39)	< 0.001
Year of schooling	3.39 (4.28)	3.52 (4.31)	2.95 (4.13)	< 0.001
Hypertension (%)	4875 (44.10)	3762 (44.00)	1113 (44.60)	0.581
DM (%)	1158 (10.50)	879 (10.30)	279 (11.20)	0.204
Heart disease (%)	2013 (18.20)	1487 (17.40)	526 (21.10)	< 0.001
Stroke or cerebrovascular disease (%)	1233 (11.20)	846 (9.90)	387 (15.50)	< 0.001
Respiratory disease (%)	1157 (10.50)	862 (10.10)	295 (11.80)	0.013
Cancer (%)	172 (0.60)	133 (0.60)	39 (1.60)	1

BMI: body mass index; DM: diabetes mellitus; hPDI: healthy plant-based dietary index; uPDI: unhealthy plant-based dietary index

### Correlation between plant-based diet indices and falls

We analyzed the connection between two plant-based diet indices and falls, using the logistic regression. The results of the logistic regression analysis were presented in Table 2. Participants in the highest quartile for hPDI showed a decreased risk of falls, with OR of 0.78 (95% CI: 0.69–0.89) compared to participants in the lowest quartile, while the highest uPDI was associated with increased odds of falls. Furthermore, we found that with 10-unit increment in hPDI, the risk of fall decreased by 15% (OR per 10-unit increment = 0.85, 95% CI: 0.79–0.91, *P*-trend < 0.001), while for every 10-unit increment in uPDI, the risk of fall went up by 21% (OR per 10-unit increment = 1.21, 95% CI:1.13–1.30, *P*-trend < 0.001).

**Table 2:** OR and 95% CI for falls, according to quartile for hPDI and uPDI

	hPDI				uPDI		
	Median	Events	OR (95% CI)		Median	Events	OR (95% CI)
Quartile 1	43	693	Ref.		42	503	Ref.
Quartile 2	49	587	0.87 (0.77–0.99)		48	555	1.07 (0.93–1.23)
Quartile 3	52	603	0.86 (0.75–0.97)		53	693	1.13 (0.99–1.30)
Quartile 4	58	610	0.78 (0.69–0.89)		58	742	1.37 (1.20–1.58)
Per 10-unit increment			0.85 (0.79–0.91)				1.21 (1.13–1.30)
*P*-trend			< 0.001				< 0.001

Data were shown as OR (95% CI) which was evaluated using logistic regression models adjusting for age, sex, ethnicity, smoking status, residence, year of schooling, alcohol consumption, marital status, body mass index. CI: confidence interval; hPDI: healthy plant-based dietary index; uPDI: unhealthy plant-based dietary index; OR: odd ratio

### Subgroup analysis between the two plant-based indices and falls

**Fig. 2:**
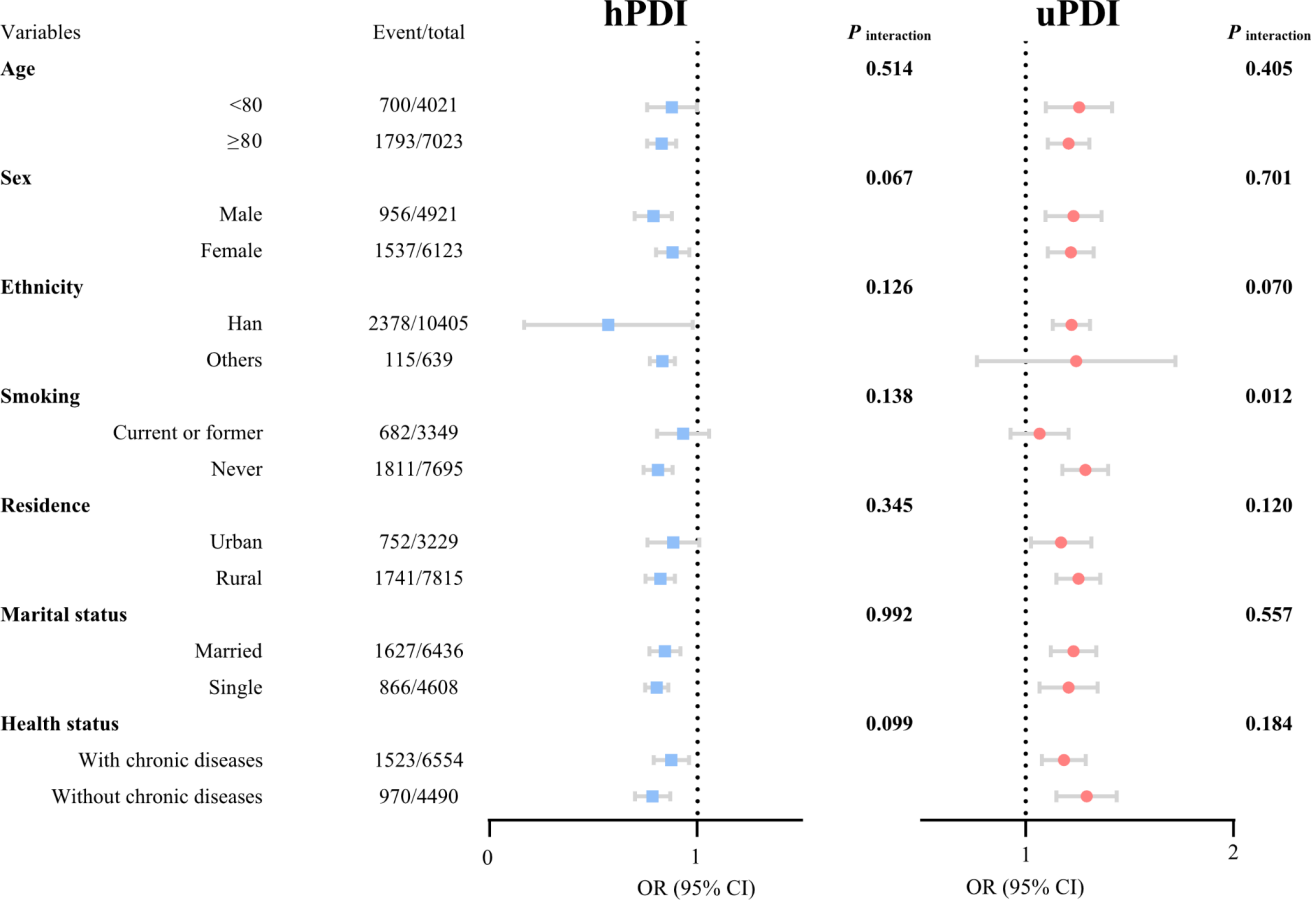
Subgroup analysis between hPDI and falls, ORs and 95% CI for fall risk per 10-unit increment in adherence to healthy plant-based diet indices. The logistic model adjusted for age, sex, ethnicity, smoking status, residence, marital status, year of schooling, alcohol consumption, body mass index. CI: confidence interval; uPDI: unhealthy plant-based diet; hPDI: healthy plant-based diet; OR: odd ratio

Figure 2 illustrated the subgroup analysis between uPDI, hPDI and falls. While investigating the association between uPDI and falls, we observed the interaction among the smoking status, showing that individuals who never smoked were more likely to experience falls (*P*_interaction_ = 0.012), as no other interactions were identified.

### Restricted cubic spline analysis between plant-based diet index scores and falls

**Fig. 3:**
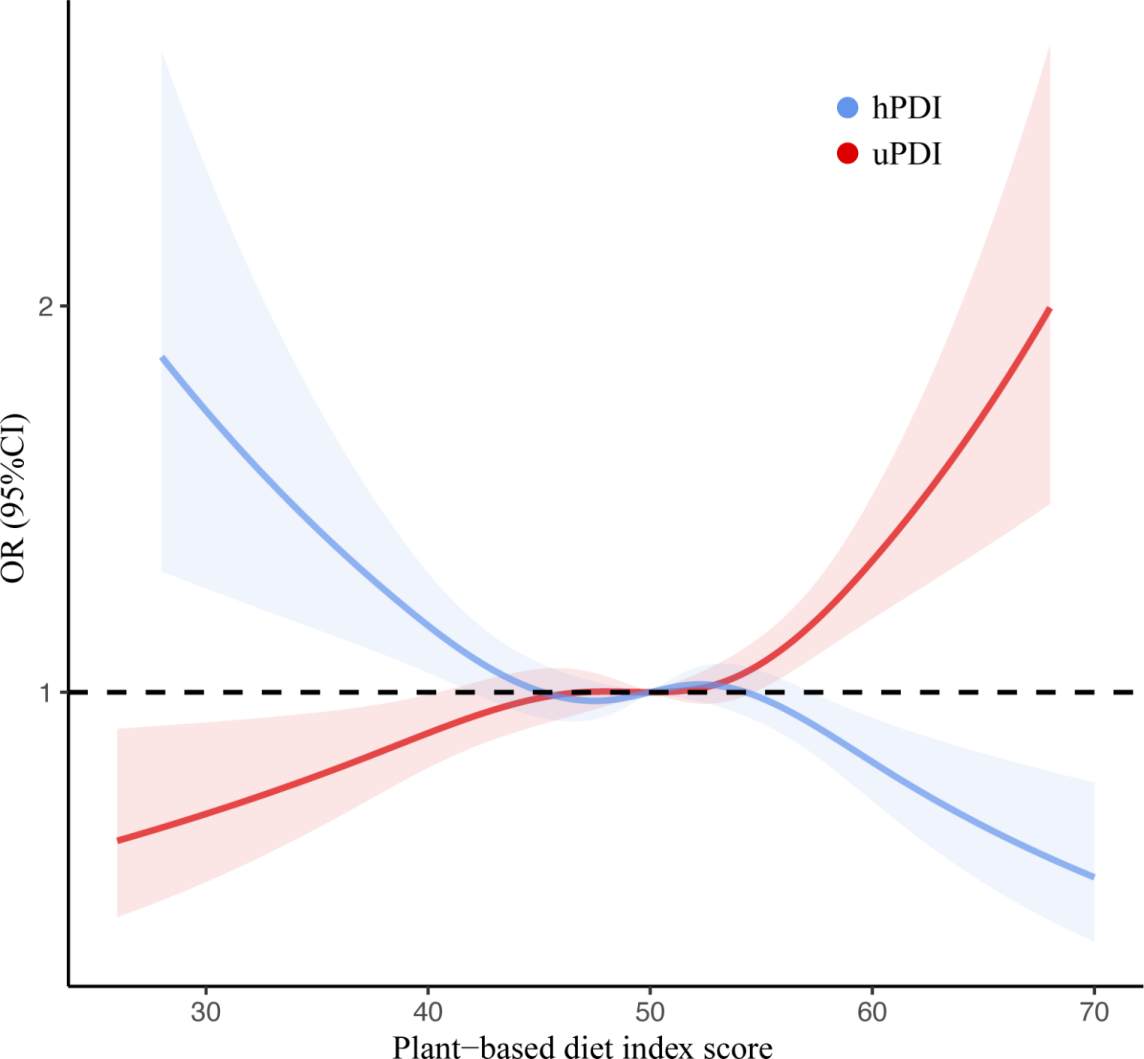
ORs and 95% CIs for fall risks, according to continuous plant-based diet index. The curves were multivariable adjusted for age, sex, ethnicity, smoking status, residence, year of schooling, alcohol consumption, marital status, body mass index. *P*_nonlinear_ for hPDI = 0.0239, *P*_nonlinear_ for uPDI = 0.0332. CI: confidence interval; hPDI: healthy plant-based diet; uPDI: unhealthy plant-based diet; OR: odd ratio

Figure 3 depicted the correlation between the plant-based diet index scores and falls applying restricted cubic splines. It demonstrated that as the plant-based diet index scores increased, the hPDI group exhibited decreased the odds of falls (*P* for overall < 0.001, *P*_nonlinear_ = 0.0239). Conversely, with the elevation of scores in the uPDI group, there was a corresponding increase in the risk of falls (*P* for overall < 0.001, *P*_nonlinear_ = 0.0332).

### Sensitivity analysis

As illustrated in Table S3, the associations between the hPDI, uPDI and falls persisted even after further adjusting for chronic diseases (hPDI: OR per 10-unit increment 0.84, 95% CI: 0.78–0.90; uPDI:1.22, 95% CI: 1.14–1.31, respectively). Likewise, after adjusting for chronic diseases, we deal with missing values through multiple imputation, and the result remained significant (hPDI: OR per 10-unit increment 0.82, 95% CI: 0.77–0.88; uPDI: 1.22, 95% CI: 1.15–1.30) (Table S4).

## Discussion

Our study demonstrated remarkable link between dietary habits and the risk of falls among older individuals. Continuously consuming higher intake of healthy plant-based food reduced the risk of falls by up to 15%. Conversely, adhering to the pattern of higher intake of animal foods and fewer healthy plant-based foods increased the incidence of falls by up to 21%. In the subgroup analysis, we observed that the population who never smoked exhibited higher rate of falls if they consumed larger amounts of animal meat and less healthy plant-based foods. Notably, the results remained consistent after sensitivity analysis.

The plant-based diet emphasized consuming diverse selection of healthy plant foods, reducing risk of chronic disease such as PD, DM, cognitive impairment, and stroke [18, 21, 30–32]. Additionally, plant-based diet also lowered the incidence of cataracts [33]. All of these diseases were connected to increased risk of falls [34], and the presence of multiple chronic disease further amplified this risk [35, 36]. By reducing the occurrence of these disease, a diet rich in healthy plant-based foods might indirectly decrease the risk of falls. Previous studies provided some evidence supporting the influence of diet on the occurrence of falls. A study [13] involving 2071 participants age 60 years and older found that a mediterranean-style dietary pattern, characterized by higher intake of vegetables and fruit, was associated with a reduced risk of falls. Another research by McTiernan [37] examined the impact of dietary patterns on fractures, and bone mineral density among postmenopausal women also demonstrated that a higher intake of grain, vegetable and fruit diet combined with low fat diet modestly decreased the risk of harmful falls. Sim’s prospective cohort study [38] also indicated that increasing vegetable diversity decreased the risk of injurious falls among older Australian women. Given these findings, it seems reasonable to suggest that older adults increase their intake of healthy plant foods to potentially reduce the incidence of falls. More detailed future work is needed to evaluate the impact of chronic diseases and diet on falls in older adults.

Vegetables and fruit were rich in numerous active ingredients, including carotenoids and vitamin E. These components might potentially decrease the risk of osteoporosis by inhibiting bone resorption and stimulating bone formation [39, 40]. In addition, plant-based diet might regulate inflammation by reducing the expression of cellular and circulating biomarkers related to various diseases. Consuming higher intake of fruit and vegetables led to decreased levels of circulating CRP, IL-6 and TNF-α. Elevated levels of these inflammatory factors were linked to reduced muscle strength and muscle mass [41, 42]. Therefore, emphasizing a diet rich in plant-based foods may offer benefits in falls by influencing on musculoskeletal health.

In our study, we observed an intriguing interaction between smoking status and the correlation between uPDI and falls. This phenomenon might be attributed to the exposure of non-smokers to secondhand smoke. More than half of non-smokers were subjected to second-hand smoke in China, inhaling high concentrations of toxic and carcinogenic compounds resulting from incomplete combustion, which surpass those inhaled by smokers [43]. The elevated levels of these harmful compounds increased the risk of various chronic diseases, including cardiovascular disease and cancer. Especially Fu’s study found that the second-hand smoke were associated with frailty and pre-frailty in the older non-smoking population [44]. All of these diseases were high-risk factors for falls. Indirectly, second-hand smoke might result in an elevated risk of falls through these health implications. It is important to acknowledge the limitation of our study. Firstly, fall was defined using a self-reported or family-reported questionnaire record, which may have led to information bias. Our data are collected using objective data collection methods such as questionnaires. The self-reporter or kin-reporter reviewed and recorded events promptly to minimize the impact of time on personal memory, thereby ensuring the authenticity and accuracy of the data. Therefore, we hope to further test the current findings with other databases. Secondly, this study was cross-sectional, which limited our ability to establish a causal relationship between PDI and the prevalence of falls in the elderly population. Cross-sectional designs capture a single point in time, making it challenging to infer temporal or causal connections. Besides, the reliance on self-reported data introduced the potential for recall bias. Participants may have inaccurately remembered or reported their dietary habits and incidences of falls, which could impact the study’s findings. This limitation is particularly relevant in studies involving older adults, who may have varying degrees of memory accuracy. Additionally, the study did not account for the potential influence of medication usage on falls [2]. Older adults often use multiple medications, which can vary widely and influence the risk of falls. This factor could have confounded our results, and future studies should consider examining the role of medication in this context. Furthermore, the results are limited to the Chinese population and may not be generalizable to other ethnic groups. Cultural differences, dietary habits, and genetic factors could influence the relationship between PDI and falls, necessitating caution when applying these findings to diverse populations.

Despite these limitations, our study provides valuable insights into the association between dietary patterns and fall risk in older adults. However, future research should address these limitations by using longitudinal designs, objective data collection methods, and considering the impact of medication use and other potential confounders. Besides, we expected to explore the potential influence of medication usage and multimorbidity status with fall. We will then combine with other databases to collect data and information from as many different sources as possible, and then compare and validate them to reduce the impact of personal biases. Our study is only a cross-sectional analysis, and we will conduct a longitudinal study with other databases to make our findings more persuasive.

## Implications for research and practice

The plant-based diet index was linked to the probability of falling among older adults, while persisting in consuming higher proportion of animal foods and less health plant foods may connect to higher rate of falling. Interestingly, in the investigation of connection between falls and consuming higher amounts of less healthy foods and animal foods, we found that the non-smokers were more prone to experiencing falls. These findings suggested that guiding the dietary choices of the older population toward healthy plant-based diet may help reduce the occurrence of falls.

## Electronic supplementary material

Below is the link to the electronic supplementary material.

**Supplementary Material 1**: **Table S1**: Plant-based diet index scoring; **Table S2**: STROBE checklist; **Table S3**: ORs and 95% CI for fall risk in adherence to different plant-based diet after further adjusting for the chronic disease; **Table S4**: ORs and 95% CI for fall risk in adherence to different plant-based diet index after multiple imputation for covariates. **Table S5**: Missing percentage of the covariates.

## Data Availability

No datasets were generated or analysed during the current study.

## Declarations

Our study was adhered strictly with the laws of China, ensuring full compliance.
