# Supplementary material for: Plant-based index linked to fall risk in older Chinese adults: cross-sectional evidence from a national cohort
Source: Aging Clin Exp Res. 2024 Sep 5;36(1):183. doi: 10.1007/s40520-024-02838-z (PMC11377554; doi:10.1007/s40520-024-02838-z)
Supplement: Supplementary file 1 — Supplementary Material 1: Table S1: Plant-based diet index scoring; Table S2: STROBE checklist; Table S3: ORs and 95% CI for fall risk in adherence to different plant-based diet after further adjusting for the chronic disease; Table S4: ORs and 95% CI for fall risk in adherence to different plant-based diet index after multiple imputation for covariates. Table S5: Missing percentage of the covariates. [file 40520_2024_2838_MOESM1_ESM.docx]

**Plant-Based Index Linked to Fall Risk in Older Chinese Adults: Cross-Sectional Evidence from a national cohort**

**Fuli Yang^1, #^, Junguo Jin^2, #^, Jieliang Liu^1^, Xiaoqi Lu^1^, Huyi Jiang^3^, Huixin Tan^3^, Fenghua Zhou^4, *^, Ping Zeng^1, 3, *^**

^1^Guangdong Cardiovascular Institute, Guangdong Provincial People's Hospital, Guangdong Academy of Medical Sciences

^2^Department of Cardiology, Peking University First Hospital, No. 8 Xishiku St, Xicheng District, Beijing, 100034, China.

^3^Department of Cardiology, Guangdong Provincial People's Hospital (Guangdong Academy of Medical Sciences), Southern Medical University

^4^School of Traditional Chinese Medicine, Southern Medical University, Guangzhou, 510515, China

#The authors contributed equally to this article

*Corresponding authors:

Fenghua Zhou

School of Traditional Chinese Medicine, Southern Medical University, Guangzhou, 510515, China

**E-mail: wendyzhou515@126.com**

Ping Zeng

Department of Cardiology, Guangdong Cardiovascular Institute, Guangdong Provincial People’s Hospital (Guangdong Academy of Medical Sciences), Southern Medical University, Guangzhou 510080, Guangdong, China

**E-mail:** [**zengping@gdph.org.cn**](mailto:zengping@gdph.org.cn)

Table S1. Plant-based diet index scoring

| **Food** | **Frequency** | **hPDI** | **uPDI** |
| --- | --- | --- | --- |
| Plant-based Food Groups | | | |
| **Healthy** | | | |
| Whole grain | Yes | 5 | 1 |
|  | No | 1 | 5 |
| Vegetable oil | Yes | 5 | 1 |
|  | No | 1 | 5 |
| Fresh fruit | Almost everyday | 5 | 1 |
|  | Quite often | 4 | 2 |
|  | Occasionally | 2 | 4 |
|  | Rarely or never | 1 | 5 |
| Fresh vegetable | Almost everyday | 5 | 1 |
|  | Quite often | 4 | 2 |
|  | Occasionally | 2 | 4 |
|  | Rarely or never | 1 | 5 |
| Legume | Almost everyday | 5 | 1 |
|  | ≥1 time/week | 4 | 2 |
|  | ≥1 time/month | 3 | 3 |
|  | Occasionally | 2 | 4 |
|  | Rarely or never | 1 | 5 |
| Garlic | Almost everyday | 5 | 1 |
|  | ≥1 time/week | 4 | 2 |
|  | ≥1 time/month | 3 | 3 |
|  | Occasionally | 2 | 4 |
|  | Rarely or never | 1 | 5 |
| Nut | Almost everyday | 5 | 1 |
|  | ≥1 time/week | 4 | 2 |
|  | ≥1 time/month | 3 | 3 |
|  | Occasionally | 2 | 4 |
|  | Rarely or never | 1 | 5 |
| Tea | Almost everyday | 5 | 1 |
|  | ≥1 time/week | 4 | 2 |
|  | ≥1 time/month | 3 | 3 |
|  | Occasionally | 2 | 4 |
|  | Rarely or never | 1 | 5 |
| **Less healthy** | | | |
| Refined grain | Yes | 1 | 5 |
|  | No | 5 | 1 |
| Sugar | Almost everyday | 1 | 5 |
|  | ≥1 time/week | 2 | 4 |
|  | ≥1 time/month | 3 | 3 |
|  | Occasionally | 4 | 2 |
|  | Rarely or never | 5 | 1 |
| Salt-preserved vegetable | Almost everyday | 1 | 5 |
|  | ≥1 time/week | 2 | 4 |
|  | ≥1 time/month | 3 | 3 |
|  | Occasionally | 4 | 2 |
|  | Rarely or never | 5 | 1 |
| **Animal-based Food Groups** | | | |
| Animal fat | Yes | 1 | 5 |
|  | No | 5 | 1 |
| Meat | Almost everyday | 1 | 5 |
|  | ≥1 time/week | 2 | 4 |
|  | ≥1 time/month | 3 | 3 |
|  | Occasionally | 4 | 2 |
|  | Rarely or never | 5 | 1 |
| Fish | Almost everyday | 1 | 5 |
|  | ≥1 time/week | 2 | 4 |
|  | ≥1 time/month | 3 | 3 |
|  | Occasionally | 4 | 2 |
|  | Rarely or never | 5 | 1 |
| Egg | Almost everyday | 1 | 5 |
|  | ≥1 time/month | 3 | 3 |
|  | Occasionally | 4 | 2 |
|  | Rarely or never | 5 | 1 |
| Dairy products | Almost everyday | 1 | 5 |
|  | ≥1 time/week | 2 | 4 |
|  | ≥1 time/month | 3 | 3 |
|  | Occasionally | 4 | 2 |
|  | Rarely or never | 5 | 1 |

Table S2. STROBE checklist

|  | Item No. | Recommendation | Page No. |
| --- | --- | --- | --- |
| **Title and abstract** | 1 | (*a*) Indicate the study’s design with a commonly used term in the title or the abstract | 1 |
|  |  | (*b*) Provide in the abstract an informative and balanced summary of what was done and what was found | 2 |
| **Introduction** |  |  |  |
| Background/rationale | 2 | Explain the scientific background and rationale for the investigation being reported | 3-4 |
| Objectives | 3 | State specific objectives, including any prespecified hypotheses | 4 |
| **Methods** |  |  |  |
| Study design | 4 | Present key elements of study design early in the paper | 4 |
| Setting | 5 | Describe the setting, locations, and relevant dates, including periods of recruitment, exposure, follow-up, and data collection | 4 |
| Participants | 6 | *Cross-sectional study*—Give the eligibility criteria, and the sources and methods of  selection of participants | 4 |
| Variables | 7 | Clearly define all outcomes, exposures, predictors, potential confounders, and effect modifiers. Give diagnostic criteria, if applicable | 5 |
| Data sources/ measurement | 8* | For each variable of interest, give sources of data and details of methods of assessment (measurement). Describe comparability of assessment methods if there is more than one group | 4-5 |
| Bias | 9 | Describe any efforts to address potential sources of bias | 5 |
| Study size | 10 | Explain how the study size was arrived at | 4 |
| Quantitative variables | 11 | Explain how quantitative variables were handled in the analyses. If applicable, describe which groupings were chosen and why | 5 |
| Statistical methods | 12 | (*a*) Describe all statistical methods, including those used to control for confounding | 5-6 |
|  |  | (*b*) Describe any methods used to examine subgroups and interactions | 6 |
|  |  | (*c*) Explain how missing data were addressed | 6 |
|  |  | (*d*) Describe any sensitivity analyses | 6 |
| Participants | 13* | (a) Report numbers of individuals at each stage of study—eg numbers potentially eligible, examined for eligibility, confirmed eligible, included in the study, completing follow-up, and analysed | 6 |
|  |  | (b) Give reasons for non-participation at each stage | 4 |
|  |  | (c) Consider use of a flow diagram | 17 |
| Descriptive data | 14* | (a) Give characteristics of study participants (eg demographic, clinical, social) and information on exposures and potential confounders | 6 |
|  |  | (b) Indicate number of participants with missing data for each variable of interest | 4 |
| Main results | 16 | Give unadjusted estimates and, if applicable, confounder-adjusted estimates and their precision (eg, 95% confidence interval). Make clear which confounders were adjusted for and why they were included | 6-7 |
| Other analyses | 17 | Report other analyses done—eg analyses of subgroups and interactions, and sensitivity analyses | 7 |
| Key results | 18 | Summarise key results with reference to study objectives | 6 |
| Limitations | 19 | Discuss limitations of the study, taking into account sources of potential bias or imprecision. Discuss both direction and magnitude of any potential bias | 9 |
| Interpretation | 20 | Give a cautious overall interpretation of results considering objectives, limitations, multiplicity of analyses, results from similar studies, and other relevant evidence | 7-9 |
| Generalisability | 21 | Discuss the generalisability (external validity) of the study results | 9 |
| Other information |  |  |  |
| Funding | 22 | Give the source of funding and the role of the funders for the present study and, if applicable, for the original study on which the present article is based | 10 |

Table S3. ORs and 95% CI for fall risk in adherence to different plant-based diet after further adjusting for the chronic disease

|  | hPDI | uPDI |
| --- | --- | --- |
|  | OR (95% CI) | OR (95% CI) |
| Quartile 1 | Ref. | Ref. |
| Quartile 2 | 0.85 (0.75-0.97) | 1.08 (0.94-1.24) |
| Quartile 3 | 0.84 (0.74-0.95) | 1.15 (1.00-1.31) |
| Quartile 4 | 0.77 (0.67-0.87) | 1.40 (1.22-1.61) |
| Per 10-unit increment | 0.84 (0.78-0.90) | 1.22 (1.14-1.31) |
| *P*-trend | <0.001 | <0.001 |

The logistic regression models adjusted for age, sex, ethnicity, smoking status, residence, year of schooling, alcohol consumption, marital status, BMI and further adjusted for hypertension, heart disease, diabetes mellitus, respiratory disease, stroke/cerebrovascular disease and cancer. CI: confidence interval; OR: odd ratio.

Table S4. ORs and 95% CI for fall risk in adherence to different plant-based diet indices after multiple imputation for covariates

|  | hPDI | uPDI |
| --- | --- | --- |
|  | OR (95% CI) | OR (95% CI) |
| Quartile 1 | Ref. | Ref. |
| Quartile 2 | 0.86 (0.77-0.95) | 1.10 (0.97-1.24) |
| Quartile 3 | 0.83 (0.75-0.93) | 1.20 (1.06-1.35) |
| Quartile 4 | 0.75 (0.67-0.84) | 1.39 (1.23-1.57) |
| Per 10-unit increment | 0.82 (0.77-0.88) | 1.22 (1.15-1.30) |
| *P*-trend | <0.001 | <0.001 |

The logistic regression models adjusted for age, sex, ethnicity, smoking status, residence, year of schooling, alcohol consumption, marital status, body mass index after multiple imputation. CI: confidence interval; OR: odd ratio.

Supplementary Table S5 Missing percentage of the covariates. (Total N = 14807)

| Covariates | Sample size before imputation | Missing percentage (%) |
| --- | --- | --- |
| Age | 14796 | 0.0 |
| Ethnic group | 14615 | 1.3 |
| Place of residence | 14792 | 0.1 |
| Marital status | 14452 | 2.4 |
| Alcohol consumption | 14689 | 0.8 |
| Smoking status | 14392 | 2.8 |
| Years of schooling | 14496 | 2.1 |
| Body mass index | 13859 | 6.4 |
| Chronic diseases | 13400 | 9.5 |
